# Comparative Metabolomic Analysis of Three Medicinal *Paphiopedilum* Species Reveals Divergence in Antioxidant Capacity and Functional Compound Profiles

**DOI:** 10.3390/molecules30142961

**Published:** 2025-07-14

**Authors:** Jinhan Sang, Yishan Yang, Kanghua Xian, Jiang Su, Jianmin Tang, Chuanming Fu, Fengluan Tang, Xiao Wei

**Affiliations:** 1 Guangxi Key Laboratory of Plant Functional Substances and Resources Sustainable Utilization, Guangxi Institute of Botany, Guilin 541006, China; a13196221635@163.com (J.S.); yangyishan0113@163.com (Y.Y.); xkhgxib@163.com (K.X.); 3374766861@163.com (J.S.); 18877384841@163.com (J.T.); fuchuanmingming@163.com (C.F.); 2 School of Ecology and Nature Conservation, Beijing Forestry University, Beijing 100083, China

**Keywords:** *Paphiopedilum*, widely targeted metabolomics, flavonoid metabolites, antioxidant activity, biosynthetic pathways

## Abstract

This study explores the metabolite diversity and potential medicinal value of three *Paphiopedilum* species—*P. dianthum*, *P. micranthum*, and *P. barbigerum*—using widely targeted metabolomics via HPLC-MS/MS in conjunction with in vitro antioxidant assays. A total of 2201 metabolites were detected across the three species, with flavonoids emerging as the dominant class (480 compounds, accounting for 21.8% of total metabolites). Comparative metabolomic analysis showed that flavonoid levels varied most prominently among the species. Notably, the metabolic profile of *P. barbigerum* (PB) diverged substantially from those of *P. dianthum* (PD) and *P. micranthum* (PM), which shared a higher degree of similarity with each other. Quantitative evaluation of antioxidant-associated metabolites revealed that PB exhibited the greatest enrichment in compounds with antioxidant potential, particularly flavonoids and phenolic acids, followed by PM and PD. These results were corroborated by antioxidant assays, in which PB demonstrated the highest free radical scavenging activity, with PM and PD displaying progressively lower effects. Differences in flavonoid content likely underpin these variations in antioxidant capacity. KEGG pathway enrichment analysis indicated that differentially expressed metabolites were primarily involved in flavonoid-associated biosynthetic routes, notably flavonoid biosynthesis (ko00941) and isoflavonoid biosynthesis (ko00943), with ko00941 being the most enriched. Within this pathway, PB showed eight significantly upregulated flavonoid metabolites, while PM and PD had seven and five, respectively. The observed differences may stem from species-specific expression of key biosynthetic enzymes such as flavonoid 3′-hydroxylase (F3′H) in PM and flavonoid 3′,5′-hydroxylase (F3′5′H) in PB, which influence both flavonoid composition and antioxidant potential.

## 1. Introduction

The genus *Paphiopedilum*, a member of the Orchidaceae family, comprises terrestrial, semi-epiphytic, and epiphytic herbaceous species that are highly prized for both their ornamental appeal and ecological importance. Distinguished by a pouch-like labellum, these plants are popularly known as “slipper orchids” [1]. Their distribution follows a classical Southeast Asian biogeographic pattern, originating in southern China and extending through the Southeast Asian mainland to various island archipelagos [2]. Globally, 109 species and 26 varieties have been documented, predominantly in tropical and subtropical regions. China, recognized as a major center of biodiversity for *Paphiopedilum*, is home to 34 native species. These are concentrated in southwestern provinces, with Yunnan (30 species), Guangxi (15 species), and Guizhou (8 species) being the primary habitats [3]. The horticultural popularity of *Paphiopedilum* is largely due to its striking floral architecture, varied colors, and prolonged blooming period (ranging from three to eight weeks), earning it a prominent position in high-end floriculture [4,5]. However, beyond their ornamental significance, several *Paphiopedilum* species are traditionally employed in herbal medicine. For instance, *Paphiopedilum barbigerum* Tang & F. T. Wang is used to clear internal heat and detoxify the body, often prescribed for carbuncles and skin infections [6]. *Paphiopedilum dianthum* Tang & F. T. Wang is traditionally used to treat splenomegaly [7], while *Paphiopedilum micranthum* Tang & F. T. Wang is believed to alleviate fever, aid in the management of measles eruptions, nourish the heart and calm the mind, and treat conditions such as pneumonia, insomnia, and neurasthenia, and is recognized for its ethnomedicinal applications [8]. The combined pressures of ornamental appeal and medicinal demand have driven intensive overcollection and habitat degradation, resulting in a dramatic decline in *Paphiopedilum* populations. Their naturally low reproductive rates and poor population recovery further exacerbate their vulnerability [9]. Consequently, many species are now classified as “Critically Endangered” on the IUCN Red List and listed in Appendix I of CITES, restricting international trade. In China’s 2021 edition of the National List of Key Protected Wild Plants, all *Paphiopedilum* species except *P. hirsutissimum* and *P. micranthum* (Class II) are granted Class I protection. Current research on *Paphiopedilum* conservation primarily addresses ex situ propagation [10,11], genetic variation [12,13], chloroplast genome analysis [14,15], and phylogenetic studies [16], while pharmacological exploration remains limited.

As endangered flora often produce unique secondary metabolites in response to their specific ecological niches, they represent untapped reservoirs of potential drug candidates. An integrated research approach encompassing molecular biology, ecological science, and pharmacognosy could uncover their medicinal value while informing conservation efforts. Sustainable strategies that combine germplasm conservation, artificial cultivation, and habitat rehabilitation—supported by financial and policy frameworks—may offer viable alternatives to wild harvesting. Such integrated conservation–utilization models have proven effective in other endangered medicinal plants. For example, *Dendrobium officinale* Kimura & Migo, once collected exclusively from the wild, is now extensively cultivated over 30,000 hectares in China, generating an annual output exceeding CNY 50 billion [17,18,19,20]. Similarly, taxol (from *Taxus* spp.) and gastrodin (from *Gastrodia elata*) exemplify successful transformations from endangered resources to industrial-scale pharmaceuticals. Advanced analytical platforms such as LC-, UPLC-, and GC-MS-based metabolomics allow for comprehensive identification and quantification of plant metabolites. Many natural compounds—especially flavonoids, polyphenols, and alkaloids—are recognized for their antioxidant activities and therapeutic roles in combating oxidative stress-related diseases, including cardiovascular disorders, neurodegenerative conditions, and cancers [21,22]. This study aims to characterize the in vitro antioxidant activities of three medicinal *Paphiopedilum* species and to compare their metabolite profiles using widely targeted metabolomics. The resulting data provide foundational insights into the medicinal value and potential resource development of these critically endangered orchids.

## 2. Results

### 2.1. Metabolic Profiling Analysis

**Figure 1:**
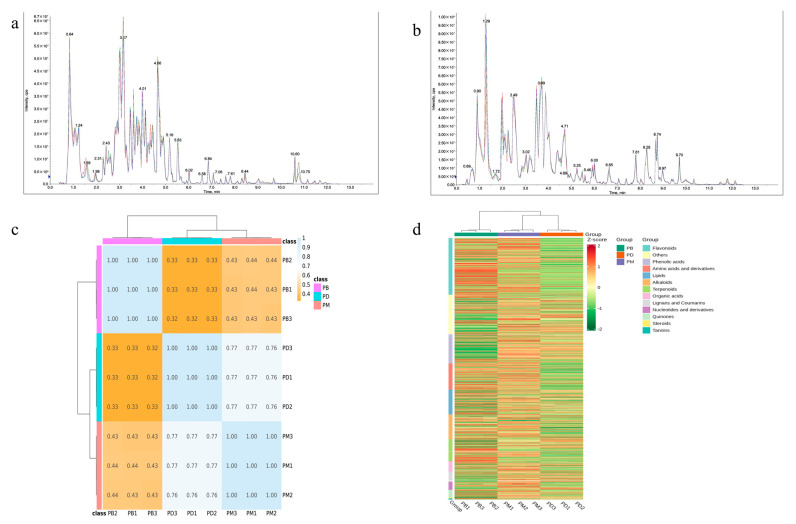
Qualitative and quantitative analysis of metabolites. (**a**,**b**): multipeak mass spectral chromatograms of metabolites acquired in negative ion mode (**a**) and positive ion mode (**b**). Different colors represent sample replicates and are used solely to distinguish overlapping chromatographic curves. (**c**): Pearson’s correlation coefficients among all samples. (**d**): cluster analysis of distinct metabolites.

In this study, HPLC-MS/MS technology was employed to systematically identify metabolites in the leaves of *Paphiopedilum barbigerum* (PB), *Paphiopedilum dianthum* (PD), and *Paphiopedilum micranthum* (PM). To evaluate the reliability of the analytical method and instrumental stability, total ion chromatogram (TIC) overlay analysis was performed on quality control (QC) samples (Figure 1a,b). The results demonstrated high overlap of metabolite detection curves in both positive and negative ion modes, with consistent retention times and signal intensities. This indicates that the established HPLC-MS/MS method exhibits excellent reproducibility and stability, enabling precise metabolomic detection in *Paphiopedilum* leaves. Cluster analysis based on Pearson correlation analysis of the three groups revealed relatively tight clustering among PB samples, while PD and PM samples showed higher correlation. This suggests that the composition and abundance of metabolites in PD and PM may be more similar compared to PB (Figure 1c). Further classification of metabolites in the three *Paphiopedilum* species (Figure 1d) demonstrated distinct metabolic accumulation patterns. Specifically, PB exhibited a metabolic abundance profile significantly different from PD and PM, implying potential divergence in metabolic regulation or environmental adaptation mechanisms. Notably, PB displayed higher abundances of flavonoids, phenolic acids, and glycosides, while PD and PM showed elevated relative abundances of certain metabolites, such as amino acid derivatives and steroids.

**Figure 2:**
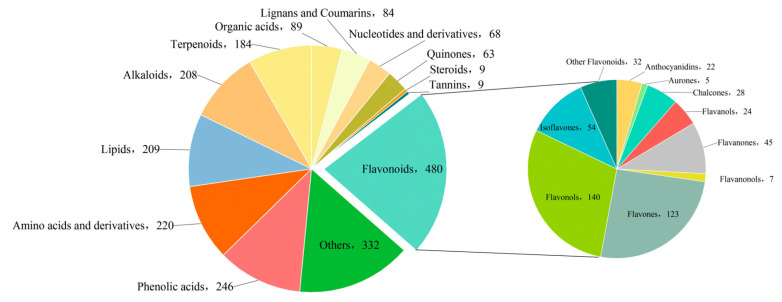
Classification of detected metabolites.

A total of 2201 metabolites spanning 13 classes were identified across the three *Paphiopedilum* species. Flavonoids constituted the largest class, with 480 metabolites (21.8%), followed by “Others” (332, 15.0%), phenolic acids (246, 11.1%), amino acids and derivatives (220, 9.9%), lipids (209, 9.4%), and alkaloids (208, 9.4%). Additionally, 184 terpenoids (8.3%), 89 organic acids (4.0%), 84 lignans and coumarins (3.8%), 68 nucleotides and derivatives (3.0%), 63 quinones (2.8%), 9 steroids (0.4%), and 9 tannins (0.4%) were detected. The 480 flavonoids were further classified into seven subclasses, with flavonols (140, 29.2%) and flavones (123, 25.6%) being the predominant groups (Figure 2).

### 2.2. PCA Analysis

**Figure 3:**
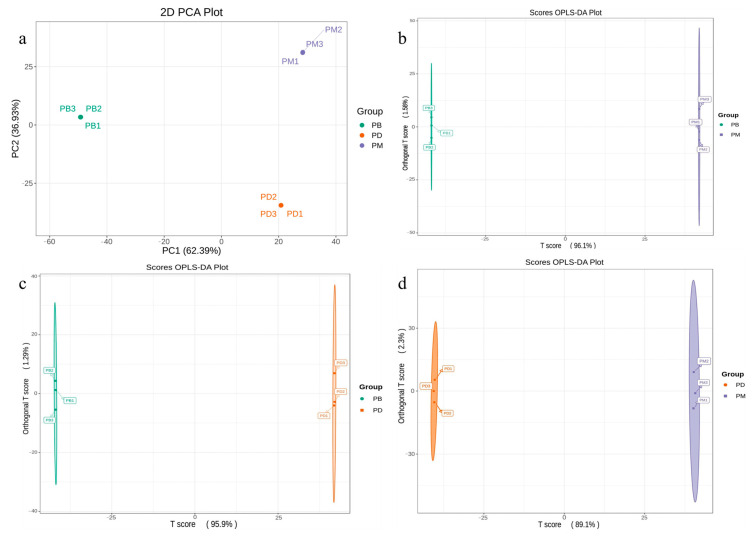
Analysis of identified metabolites. (**a**): PCA score plot. (**b**–**d**): OPLS-DA model plots for the comparison groups PB vs. PM (**b**), PB vs. PD (**c**), and PD vs. PM (**d**).

To further investigate inter- and intra-group differences among the *Paphiopedilum* species, principal component analysis (PCA) was performed on nine samples based on the relative abundance of metabolites (Figure 3a). The results revealed clear separation among the groups. The first principal component (PC1) accounted for 62.39% of the total variance, while PC2 explained 36.93%, collectively capturing 99.32% of the total variance. This indicates that the PCA effectively captured major metabolic differences among the samples. The PB group clustered predominantly on the left side, distinct from the other two groups, suggesting significant divergence in its metabolic profile along PC1. In contrast, PD and PM groups were distributed on the right side, with their separation primarily driven by PC2. This implies that metabolic differences between PD and PM differ from those distinguishing PB. Combined with the metabolic variations observed in Figure 1d, we hypothesize that differential accumulation of flavonoids and phenolic acids may account for the distinct separation of PB from PD and PM along PC1. Meanwhile, the higher levels of amino acid derivatives and alkaloids in PD, along with elevated terpenoids and lipids in PM, likely contributed to their separation along PC2.

To further explore metabolic differences among the groups, orthogonal projections to latent structures discriminant analysis (OPLS-DA) models were applied to screen the identified metabolites. All three comparative groups (PD vs. PB, PM vs. PB, PM vs. PD) exhibited robust model performance, with R^2^X > 0.7, R^2^Y > 0.7, and Q^2^ > 0.9. These results confirm the high goodness of fit and predictive reliability of the OPLS-DA models, supporting their use in subsequent differential metabolite screening.

### 2.3. Screening of Differential Metabolites

**Figure 4:**
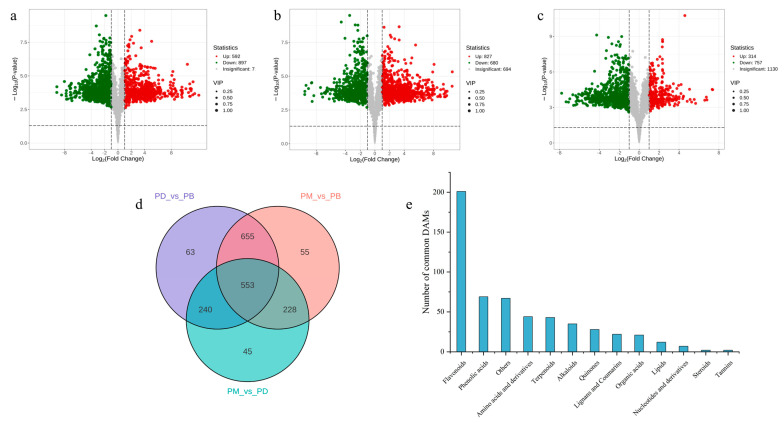
Screening of differential metabolites. (**a**–**c**): volcano plot of DAMs in PB vs. PD (**a**), PB vs. PM (**b**), and PM vs. PD (**c**). (**d**): Venn diagram showing the overlap of DAMs among PB vs. PD, PD vs. PB, and PM vs. PD. (**e**): bar chart showing the classification of shared DAMs.

Differentially accumulated metabolites (DAMs) across comparative groups were screened using thresholds of fold change ≥2 or ≤0.5 and variable importance in projection (VIP) > 1. A substantial number of DAMs were identified in pairwise comparisons: 1489 DAMs (67.7% of total metabolites) in PD vs. PB, with downregulated metabolites (897, 60.2%) significantly outnumbering upregulated ones (592, 39.8%), indicating higher overall metabolite abundance in PB compared to PD (Figure 4a). In PB vs. PM, 1507 DAMs (68.5%) were detected, with more upregulated metabolites (827, 54.9%) than downregulated (680, 45.1%), suggesting greater metabolite accumulation in PB relative to PM (Figure 4b). For PM vs. PD, 1071 DAMs (48.7%) were identified, dominated by downregulated metabolites (757, 70.7%) versus upregulated (314, 29.3%), reflecting higher metabolite abundance in PM compared to PD (Figure 4c)**.** Collectively, PB exhibited higher overall metabolite levels than PD and PM, while PM surpassed PD in metabolite abundance.

To elucidate similarities and differences in metabolite accumulation patterns, the number of DAMs across groups was visualized (Figure 4d,e). Unique DAMs were identified for each comparison: 63 in PD vs. PB, 55 in PM vs. PB, and 45 in PM vs. PD (Figure 4d). Notably, PD vs. PB and PM vs. PB shared 655 metabolites, far exceeding those shared by PM vs. PD (228) or PD vs. PB (240) (Figure 4d)**.** This further supports the pronounced divergence of PB’s metabolic profile from PD and PM, compared to the relatively smaller differences between PD and PM. However, the limited number of unique DAMs per group implies that, despite significant overall metabolic distinctions among the three *Paphiopedilum* species, a large proportion of metabolites are shared across two or more groups, potentially reflecting partial metabolic conservation within the genus. Among the 553 metabolites showing significant changes across all three species, flavonoids constituted the largest class (201), followed by phenolic acids (69) and “Others” (67).

### 2.4. Analysis of Potential Antioxidant Metabolites

To investigate the distribution patterns and relative abundance changes in antioxidant metabolites among the *Paphiopedilum* species, a systematic analysis was conducted on flavonoids, phenolic acids, terpenoids, and alkaloids within the differentially accumulated metabolites (DAMs) using metabolomic data (Table 1). In the PD vs. PB comparison, 854 antioxidant DAMs were identified, with 332 (38.9%) upregulated and 522 (61.1%) downregulated, indicating higher overall antioxidant metabolite abundance in PB compared to PD. For PM vs. PB, 837 antioxidant DAMs were detected, of which 366 (43.7%) were upregulated and 471 (56.3%) downregulated, further supporting PB’s superior antioxidant metabolite accumulation relative to PM. In the PM vs. PD comparison, 588 antioxidant DAMs were identified, with 413 (70.2%) upregulated and 175 (29.8%) downregulated, suggesting higher antioxidant metabolite levels in PM than PD. Flavonoids dominated across all comparisons. PB exhibited overall higher abundances of flavonoids compared to PD and PM, followed by phenolic acids, terpenoids, and alkaloids. Collectively, PB showed greater enrichment in multiple antioxidant metabolite classes, with superior accumulation levels relative to PD and PM, while PM surpassed PD in metabolite content.

### 2.5. Antioxidant Activity Analysis

Metabolomic analysis revealed flavonoids as the predominant differential metabolites among the three *Paphiopedilum* species, with relative abundances ranked as PB > PM > PD. To further validate and explore the functional divergence of these metabolites, in vitro antioxidant assays were performed on total flavonoid extracts from the three species.

In DPPH radical scavenging assays (Table 2), PB and PM exhibited comparable activity, with IC_50_ values of 0.194 mg/mL and 0.204 mg/mL, respectively, both significantly outperforming PD (IC_50_ = 0.490 mg/mL). This indicates stronger reducing capacities of PB and PM against DPPH radicals. For superoxide anion (O_2_^−^·) scavenging activity, PB demonstrated the highest activity (IC_50_ = 0.060 mg/mL), slightly surpassing PM (IC_50_ = 0.068 mg/mL), while PD showed the weakest activity (IC_50_ = 0.082 mg/mL). These results suggest that PB may contain higher levels or more efficient O_2_^−^· scavenging components. In hydroxyl radical (·OH) scavenging assays, PB displayed the strongest activity (IC_50_ = 0.044 mg/mL), significantly exceeding PM (IC_50_ = 0.052 mg/mL) and PD (IC_50_ = 0.054 mg/mL), implying the presence of highly effective ·OH-inhibiting constituents in PB. Collectively, PB exhibited the strongest in vitro antioxidant capacity, followed by PM, with minimal differences between them, yet both were markedly superior to PD.

### 2.6. Enrichment Analysis and KEGG Pathways

**Figure 5:**
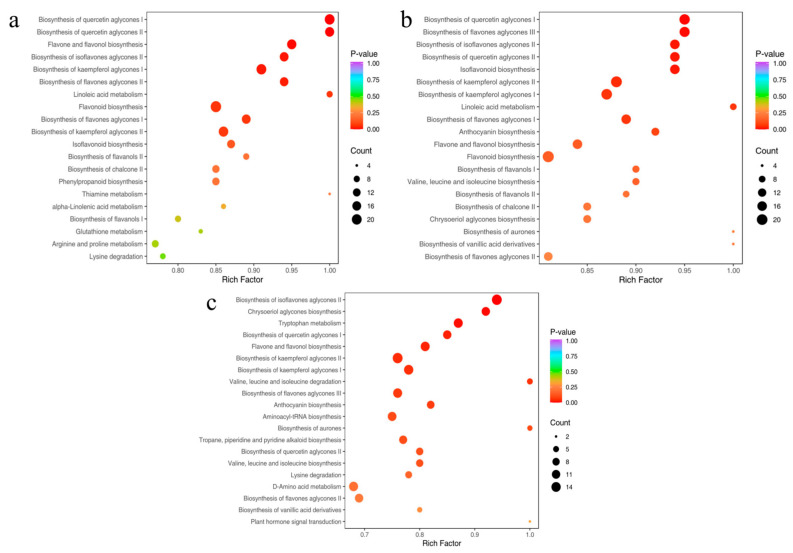
KEGG enriched pathways of PD vs. PB (**a**), PM vs. PB (**b**), and PM vs. PD (**c**).

To elucidate differences in secondary metabolic regulation among the *Paphiopedilum* species, KEGG enrichment analysis was performed on DAMs from the three comparative groups. The top 20 pathways ranked by *p*-value were visualized (Figure 5a–c). In the PD vs. PB group, DAMs were predominantly enriched in 14 flavonoid-related pathways, including biosynthesis of quercetin aglycones I (MetMap115), biosynthesis of quercetin aglycones II (MetMap116), flavone and flavonol biosynthesis (ko00944), flavonoid biosynthesis (ko00941), and isoflavonoid biosynthesis (ko00943) (Figure 5a). This highlights the pronounced metabolic divergence in flavonoids between PD and PB. Among these, flavonoid biosynthesis (ko00941) exhibited the highest number of enriched DAMs, followed by biosynthesis of quercetin aglycones I (MetMap115) and biosynthesis of kaempferol aglycones I (MetMap113).

Similarly, in the PM vs. PB group, DAMs were enriched in 17 flavonoid-related pathways, such as biosynthesis of quercetin aglycones I, biosynthesis of flavones aglycones III, isoflavonoid biosynthesis, and flavonoid biosynthesis (Figure 5b). Flavonoid biosynthesis (ko00941) again showed the highest DAM enrichment, followed by biosynthesis of kaempferol aglycones II (MetMap114). In contrast, PM vs. PD DAMs were enriched in only 11 flavonoid-related pathways, including biosynthesis of isoflavones aglycones II, biosynthesis of quercetin aglycones I, and flavone and flavonol biosynthesis (Figure 5c), with fewer enriched DAMs compared to other groups.

### 2.7. Identification of Core Metabolic Pathways

**Figure 6:**
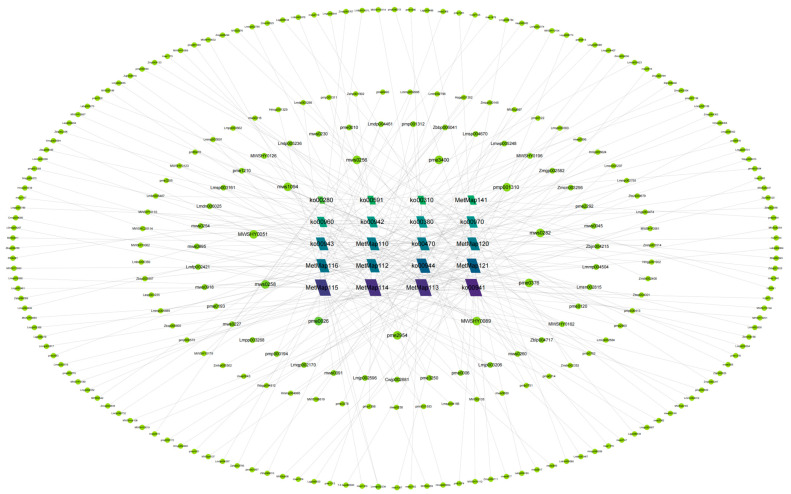
Pathway–metabolite network diagram.

After integrating the top 20 KEGG pathways from all comparative groups, the 20 most significantly enriched pathways (based on *p*-value) were selected. Metabolic network interaction analysis of associated DAMs revealed flavonoid biosynthesis (ko00941) as the core pathway driving metabolic divergence among the three *Paphiopedilum* species (Figure 6). This pathway encompassed 22 DAMs, including two phenolic acids. Additional flavonoid synthesis pathways, such as biosynthesis of kaempferol aglycones I (MetMap113), biosynthesis of kaempferol aglycones II (MetMap114), and biosynthesis of quercetin aglycones I (MetMap115), were also significantly enriched. Using degree centrality in the network analysis, L-valine (mws0256) emerged as the most central DAM, interacting with pathways including ko00970 (aminoacyl-tRNA biosynthesis), ko00960 (tropane, piperidine, and pyridine alkaloid biosynthesis), ko00310 (lysine degradation), and ko00470 (D-amino acid metabolism).

**Figure 7:**
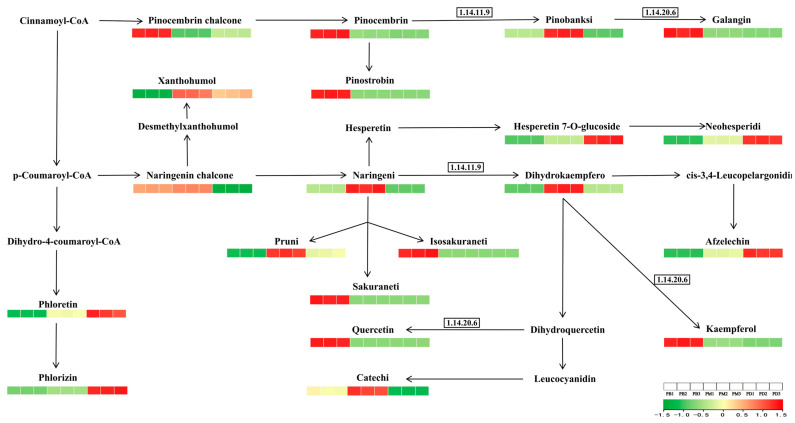
Expression of differential metabolites in the flavonoid biosynthesis pathway (ko00941).

In-depth analysis of flavonoid biosynthesis (ko00941) was performed to explore the basis of flavonoid divergence, following which the biosynthetic pathway was analyzed (Figure 7). In PB, eight metabolites—quercetin, isosakuranetin, sakuranetin, kaempferol, and others—were significantly upregulated. PM showed upregulation of seven metabolites, including naringenin, aromadendrin, and catechin, while PD exhibited upregulation of only five metabolites, such as phloretin and phlorizin. Notably, enzyme activity levels likely influenced metabolite abundance. Flavonoid 3′-monooxygenase (F3′H, EC 1.14.11.9) was hypothesized to be highly expressed in PM, whereas flavonoid 3′,5′-hydroxylase (F3′5′H, EC 1.14.20.6) showed potential overexpression in PB. Comparative analysis suggested that F3′5′H may contribute more significantly to antioxidant capacity, correlating with PB’s superior antioxidant performance.

## 3. Discussion

### 3.1. Flavonoids and Phenolic Acids May Serve as Primary Drug-like Components

While *Paphiopedilum* is best known for its ornamental beauty, its medicinal potential akin to other members of the Orchidaceae family merits deeper investigation. In the present study, a widely targeted metabolomic approach was applied to three endangered *Paphiopedilum* species to systematically catalog their secondary metabolite profiles. Results revealed that flavonoids and phenolic acids collectively constituted the most abundant and significant metabolite classes, with 480 compounds representing 21.8% of all detected metabolites. These classes are widely acknowledged for their strong antioxidant properties, making them critical indicators of medicinal potential. Compared to synthetic antioxidants, plant-derived compounds such as flavonoids and phenolic acids generally offer superior biocompatibility, lower toxicity, greater efficacy, and improved bioavailability [23]. For instance, dietary flavonoids like lycopene and lutein have demonstrated cardiovascular protective effects by attenuating oxidative stress and inhibiting atherosclerotic plaque formation [24]. Similarly, catechins and caffeic acid, which are commonly found in green tea, have been implicated in cancer chemoprevention and adjunctive cancer therapies due to their antioxidative mechanisms [25].

*P. barbigerum* (PB) has been traditionally employed to treat carbuncles and boils—conditions that align with what modern clinical practice categorizes as “surgical ulcers.” These disorders typically result from heat-induced toxins, moisture accumulation, insect stings, or traumatic infections, culminating in skin and soft tissue infections. Contemporary medical studies have demonstrated that such infections are commonly bacterial in nature, involving both inflammation and oxidative stress. During infection, immune cells generate high levels of reactive oxygen species (ROS) to combat pathogens; however, excessive ROS can damage surrounding healthy tissues, impede the healing process, and intensify inflammation [26,27]. Antioxidant treatments have shown efficacy in mitigating oxidative tissue injury, modulating immune responses, and improving outcomes in cutaneous infections [28,29]. Given this context, the high levels of flavonoids and phenolic acids in PB—compounds known for their potent antioxidant activity—likely form the biochemical foundation for its traditional medicinal use. These constituents not only neutralize ROS and reduce inflammation but may also enhance immune defenses at the infection site and facilitate tissue regeneration through multi-target biological pathways, underscoring their clinical potential. *P. dianthum* (PD) is traditionally used to manage hepatosplenomegaly, a condition often indicative of liver fibrosis or cirrhosis. A key mechanism in liver fibrogenesis is lipid peroxidation [30], and antioxidants that counteract peroxidative damage are recognized as vital in preventing hepatic fibrosis [31]. Therefore, the substantial flavonoid and phenolic acid content in PD may underlie its liver-protective effects, marking these secondary metabolites as promising candidates for hepatoprotective drug development. *P. micranthum* (PM) has been historically utilized to treat conditions such as measles, pneumonia, and neurasthenia. Measles, caused by the measles virus, provokes substantial ROS production, leading to oxidative stress, tissue injury, and compromised immune cell function [32,33]. This cascade can result in serious complications, including pneumonia and encephalitis. Antioxidants have been recognized for their therapeutic potential in both managing measles and facilitating recovery after infection. PM’s medicinal value likely stems from its rich content of flavonoids and phenolic acids, which not only exert antioxidant effects but also participate in complex, multi-target mechanisms—such as anti-inflammatory, immunomodulatory, and cytoprotective actions—critical for disease treatment and recovery.

### 3.2. Flavonoids May Drive Interspecific Differences in Antioxidant Capacity

Metabolite profiling across the three *Paphiopedilum* species revealed 553 significantly altered metabolites, among which flavonoids (201 compounds) constituted the largest group, followed by phenolic acids (69) and various other classes (67). PB exhibited the highest levels of flavonoids and phenolic acids, with PM showing intermediate levels and PD the lowest. While PM and PD shared a higher degree of metabolic similarity, PM diverged more markedly from PB, suggesting possible adaptation-related metabolic differentiation. These variations likely reflect environmental pressures. Leaves, which form the primary interface with the external environment, play a central role in stress sensing and response, often relying on secondary metabolites like flavonoids and phenolic acids for protection [34]. PB thrives in shaded, rocky limestone terrains at altitudes between 800–1500 m, where sparse soil and limited water contribute to frequent drought stress [35]. In contrast, PD and PM typically grow in limestone habitats under evergreen broadleaf forests or shrubs, benefiting from humus-rich substrates. In response to environmental stress, plants commonly accumulate ROS, which, if unchecked, causes oxidative damage. To counteract this, they often elevate the production of antioxidant-rich secondary metabolites to bolster resilience. For example, Kwansi et al. [36] reported an increase in sesaminol triglucoside, which is a potent antioxidant, in sesame seeds under drought stress. Likewise, higher levels of flavonoids and carotenoids have been linked to enhanced oxidative stress resistance [37], which could explain the observed differences in secondary metabolite profiles among the *Paphiopedilum* species.

Quantitative comparisons ranked antioxidant metabolite abundance in the order PB > PM > PD, a pattern that mirrored results from in vitro antioxidant activity assays. PB exhibited the most potent broad-spectrum antioxidant effects. While PM displayed slightly lower overall antioxidant capacity, it matched PB in terms of DPPH and superoxide anion (O_2_^−^·) scavenging performance, particularly excelling in superoxide elimination. This similarity may be attributed to the presence of structurally analogous flavonoid compounds, which is supported by statistical analyses of differential metabolite content.

### 3.3. Key Enzyme Expression Differences Drive Flavonoid Metabolic Divergence

KEGG pathway annotation of the differential metabolites highlighted significant enrichment in flavonoid biosynthesis pathways across all interspecies comparisons. These pathways are well-known for their roles in plant stress responses, development, and adaptive differentiation. For instance, flavonoid and isoflavonoid biosynthesis has been identified as central to the antioxidant and pharmacological functions of legumes [38]. Similarly, flavonoid pathways contribute to drought resistance in cotton and cold adaptation in oil palm by modulating downstream antioxidant and signaling mechanisms [39,40]. These examples suggest that the flavonoid biosynthesis network in *Paphiopedilum* likely integrates environmental stimuli, offering new strategies for both optimized cultivation via controlled stress exposure and conservation based on the adaptive value of flavonoid compounds. Comparative KEGG enrichment analyses pinpointed quercetin and flavone aglycone synthesis pathways as core contributors. Quercetin and its methylated derivatives not only contribute to floral pigmentation but also enhance antioxidant defense and UV protection [41]. Their accumulation is influenced by environmental cues such as light and temperature, identifying them as key mediators of ecological adaptability [42]. In the PM vs. PD comparison, differential metabolite enrichment occurred within non-canonical pathways such as isoflavone aglycone II and quercetin aglycone I, without significant enrichment in traditional ko00941/00944 pathways. This implies the potential involvement of alternative or niche-specific flavonoid biosynthetic routes, possibly reflecting habitat-driven metabolic specialization. Prior studies have similarly reported species-specific activation of flavonoid pathways under distinct environmental stresses [43].

Among these, the canonical flavonoid biosynthesis pathway (ko00941) emerged as the central metabolic axis. PB exhibited eight upregulated flavonoid metabolites, while PM exhibited seven and PD exhibited five. These differences in metabolite abundance may be attributable to variation in the expression levels of key biosynthetic enzymes. For example, the elevated levels of quercetin and kaempferol in PB may result from heightened expression of the F3′5′H enzyme [44], which catalyzes 3′,5′-dihydroxylation of the B-ring, facilitating the formation of trihydroxylated flavonoids such as delphinidin and myricetin. In contrast, the increased flavonoid content in PM may be associated with enhanced activity of F3′H, which supports 3′-monohydroxylation to produce compounds like quercetin [45]. Since higher hydroxylation enhances antioxidant potential, these enzymatic differences likely underpin PB’s superior performance in antioxidant assays [44].

## 4. Materials and Methods

### 4.1. Materials

Healthy and mature specimens of *P. barbigerum*, *P. dianthum*, and *P. micranthum* were collected in June 2024 from wild populations in the Yachang Orchid National Nature Reserve, Baise, China. For antioxidant activity assays, ten robust disease- and pest-free individuals were randomly selected for each species. From each plant, one to two fully expanded leaves were sampled with minimal disturbance to plant growth. For metabolomic analysis, three healthy, pest-free individuals per species were randomly chosen, and a single mature leaf was collected from each plant. *P. barbigerum* was typically found growing in shaded limestone crevices, while *P. dianthum* and *P. micranthum* were collected from humus-rich rocky substrates under forest canopy. Taxonomic identification was conducted by Prof. Xiao Wei (Guangxi Institute of Botany, Chinese Academy of Sciences), and voucher specimens have been deposited in the Herbarium of the Guangxi Institute of Botany. Leaf samples designated for metabolomic analysis (1–2 cm segments) were thoroughly cleaned, flash-frozen in liquid nitrogen, and stored at −80 °C until further processing. All sampling procedures strictly adhered to CITES regulations, IUCN guidelines, and China’s National List of Key Protected Wild Plants (2021).

### 4.2. In Vitro Antioxidant Assays

#### 4.2.1. Sample Preparation

Fresh leaves of the three *Paphiopedilum* species were thoroughly washed, gently dried to remove surface moisture, and subsequently oven-dried at 60 °C for 48 h. The dried samples were then pulverized and passed through a 60-mesh sieve to obtain uniform powders for antioxidant activity assays. Precisely 0.5 g of each powdered sample was weighed into test tubes, followed by the addition of 15 mL of 70% (*v*/*v*) ethanol. The mixture was subjected to ultrasonic extraction at 60 °C and 300 W for 30 min. The samples were centrifuged at 4000 r/min for 10 min to collect the supernatant. The residue was re-extracted with 15 mL of 70% (*v*/*v*) ethanol under the same ultrasonic conditions. The supernatants from both extractions were combined and diluted to 50 mL with 70% (*v*/*v*) ethanol in a volumetric flask to obtain the test solution. Each *Paphiopedilum* species was processed in triplicate.

#### 4.2.2. DPPH Radical Scavenging Assay

The DPPH radical scavenging capacity of three *Paphiopedilum* species was determined using a modified method [46]. A 4.0 mL aliquot of the sample extract at various concentration gradients was transferred into a 10 mL centrifuge tube, followed by the addition of 4.0 mL of DPPH solution (0.1 mmol/L). The mixture was thoroughly vortexed and then incubated at room temperature in the dark for 30 min. The absorbance was measured at 517 nm, using the corresponding concentration of sample extract as the reference (*A*_1_). *A*_2_ was determined by replacing the sample extract with 4.0 mL of sample solvent, while *A*_3_ was obtained by replacing the extract with distilled water. All measurements were performed in triplicate for each treatment.

$$\mathrm{DPPH}\;(\%)=(1-\frac{A_{1}-A_{2}}{A_{3}})\times 100$$

#### 4.2.3. Hydroxyl Radical (·OH) Scavenging Assay

The hydroxyl radical (·OH) scavenging activity of three *Paphiopedilum* species was determined using a modified method [47]. A 2.0 mL aliquot of the sample extract at various concentration gradients was transferred into a 10 mL centrifuge tube. Subsequently, 1.0 mL of 2.5 mmol/L salicylic acid solution and 1.0 mL of 5.0 mmol/L ferrous sulfate solution were added, followed by the addition of 2.0 mL of deionized water. The mixture was thoroughly vortexed, and 1.0 mL of 5.0 mmol/L hydrogen peroxide solution was then added to initiate the reaction. The tubes were incubated in a water bath at 37 °C for 30 min. After incubation, the absorbance was measured at 510 nm. *A*_4_ represented the absorbance of the complete reaction system. *A*_5_ was measured by replacing 1.0 mL of hydrogen peroxide solution with deionized water, while *A*_6_ was obtained by replacing 2.0 mL of the sample extract with deionized water. All measurements were conducted in triplicate for each treatment.

$$\mathrm{OH}\;(\%)=(1-\frac{A_{4}-A_{5}}{A_{6}})\times 100$$

#### 4.2.4. Superoxide Radical (O_2_^−^·) Scavenging Assay

Similarly, the superoxide radical (O_2_^−^·) scavenging capacity was assessed with an optimized protocol [47]. A 4.5 mL aliquot of Tris-HCl buffer and 2.5 mL of deionized water were transferred into a 20 mL centrifuge tube and incubated in a water bath at 25 °C for 20 min. Subsequently, 1.5 mL of the sample extract at various concentration gradients and 0.5 mL of 25 mmol/L pyrogallol solution were added. The mixture was thoroughly vortexed and incubated again in a water bath at 25 °C for 8 min. Finally, 0.1 mmol/L HCl was added to terminate the reaction. The absorbance was measured at 320 nm using the corresponding sample solvent as the reference (*A*_7_). *A*_8_ was determined by replacing 0.5 mL of pyrogallol solution with deionized water, and *A*_9_ was obtained by replacing 1.5 mL of the sample extract with deionized water. All measurements were performed in triplicate for each treatment.

$${O_{2}}^{-}\cdot \;(\%)=(1-\frac{A_{7}-A_{8}}{A_{9}})\times 100$$

### 4.3. Metabolic Analysis

#### 4.3.1. Sample Pretreatment

The leaf samples were freeze-dried (Scientz-100F, SCIENTZ, Ningbo, China) and ground to powder (MM 400, Retsch, Haan, Germany, 30 Hz, 1.5 min). Then, the powder of the leaves (50 mg for each) was dissolved in 1.2 mL of a 70% methanol solution, respectively. The metabolites were extracted through vortexing (a total of 6 times, vortexed for 30 s every 30 min). After centrifugation (12,000 rpm, 3 min), the supernatant was filtered through a 0.22 µm microporous membrane (polyvinylidene fluoride, PVDF; Millipore, Burlington, MA, USA) and stored in an injection bottle for subsequent UPLC-MS/MS analysis. To ensure the reproducibility of the mass spectrometric (MS) results, an equal mixture of the leaf samples was used as a quality control (QC) sample.

#### 4.3.2. UPLC Conditions and ESI-MS/MS System Conditions

The sample extracts were analyzed using an UPLC-ESI-MS/MS system (UPLC, ExionLC™ AD, SCIEX, Framingham, MA, USA, https://sciex.com.cn/) and Tandem mass spectrometry system (https://sciex.com.cn/). The analytical conditions were as follows: for UPLC, Agilent SB-C18 column (Agilent Technologies, Santa Clara, CA, USA, 1.8 µm, 2.1 mm × 100 mm); the mobile phase consisted of solvent A, pure water with 0.1% formic acid, and solvent B, acetonitrile with 0.1% formic acid. Sample measurements were performed with a gradient program that employed the starting conditions of 95% A and 5% B. Within 9 min, a linear gradient to 5% A and 95% B was programmed, and a composition of 5% A and 95% B was kept for 1 min. Subsequently, a composition of 95% A and 5.0% B was adjusted within 1.1 min and kept for 2.9 min. The flow velocity was set as 0.35 mL per minute; the column oven was set to 40 °C; and the injection volume was 2 μL. The effluent was alternatively connected to an ESI-triple quadrupole-linear ion trap (QTRAP)-MS.

The ESI source operation parameters were as follows: source temperature of 550 °C; ion spray voltage (IS) of 5500 V (positive ion mode)/−4500 V (negative ion mode); ion source gas I (GSI), gas II (GSII), and curtain gas (CUR) were set at 50, 60, and 25 psi, respectively; the collision-activated dissociation (CAD) was high. QQQ scans were acquired as MRM experiments with collision gas (nitrogen) set to medium. DP (declustering potential) and CE (collision energy) for individual MRM transitions were performed with further DP and CE optimization. A specific set of MRM transitions were monitored for each period according to the metabolites eluted within this period.

#### 4.3.3. Qualitative and Quantitative Determination of Secondary Metabolites

The raw data were processed using Analyst software (v.1.6.3), and the metabolites were qualitatively analyzed based on the plant-specific MetWare metabolism database (MWDB). A quantitative analysis was conducted using the MRM mode of triple quadrupole mass spectrometry. The mass spectrometry file was opened using the MultiQuant software (v.3.0.3, AB Sciex, Framingham, MA, USA), which facilitated the integration and correction of the chromatographic peaks. The relative abundance of each metabolite was determined based on the integrated area of its corresponding chromatographic peak.

#### 4.3.4. Metabolome Data Processing and Analysis

An unsupervised principal component analysis (PCA) of the metabolites was conducted using the PCA tool on the Maiwei Cloud platform (https://cloud.metware.cn/) to elucidate distinctions in metabolites both between and within the three species. Pearson’s correlation coefficient (r) was calculated using the cor function in the R package. A coefficient with an absolute value (|r|) close to 1 indicates a strong correlation between the duplicate samples. The relative abundance data of metabolites were scaled using unit variance scaling prior to heatmap generation, which was constructed using the ComplexHeatmap package (v.2.12.1) in R (v.4.2.2).

Differential metabolites were identified using supervised multiple regression orthogonal partial least squares discriminant analysis (OPLS-DA). The screening criteria for differential metabolites were a variable importance in projection (VIP) value ≥ 1 and Log_2_|fold change| ≥ 1. The Maiwei Cloud platform (https://cloud.metware.cn/) was also used to visualize the differential metabolites between the three species of golden camellia. Based on the quantitative metabolite data for each group, the top 20 differential metabolites in each comparison group were selected based on log_2_FC. These differential metabolites were further used for network pharmacology analysis.

Based on KEGG pathway enrichment analysis, the top 20 significantly ranked metabolic pathways in each comparative group were selected for visualization using the *p*-value as the screening criterion. After integrating the KEGG enrichment results across all comparative groups, the top 20 comprehensively ranked metabolic pathways were further analyzed to elucidate potential metabolic regulatory mechanisms. Significantly enriched differential metabolites from these 20 pathways were extracted, and a metabolic network was constructed using Cytoscape_v3.9.1 (Institute for Systems Biology, Seattle, WA, USA). Topological analysis was performed to identify key metabolic nodes and their interaction relationships, thereby revealing the core structure of the metabolic regulatory network and its underlying biological significance.

## 5. Conclusions

Utilizing HPLC-MS/MS-based widely targeted metabolomics, a total of 2201 metabolites spanning 13 major classes were herein identified across three *Paphiopedilum* species. Flavonoids represented the most prominent group, accounting for 480 compounds (21.8% of total metabolites). Cluster analyses demonstrated tight sample grouping within PB, while PM and PD showed higher intergroup similarity. Differentially accumulated metabolites totaled 1489 (PD vs. PB), 1507 (PB vs. PM), and 1071 (PD vs. PM), with overall metabolite abundance following the trend PB > PD > PM. Flavonoids were the dominant contributors to these differences. PB exhibited the highest in vitro antioxidant activity, as measured by DPPH, superoxide anion, and hydroxyl radical scavenging, followed by PM and PD. This activity correlated with the relative abundance of flavonoids and phenolic acids in each species. KEGG enrichment analysis identified flavonoid biosynthesis (ko00941) and isoflavonoid biosynthesis (ko00943) as core metabolic pathways. The observed upregulation of flavonoid metabolites in PB and PM, particularly relative to PD, suggests that differential expression of F3′5′H in PB and F3′H in PM may drive species-specific differences in antioxidant capacity.

## Figures and Tables

**Table 1:** Statistics of the DAMs with putative antioxidants in comparison groups.

Class	PD vs. PB		PM vs. PB		PM vs. PD	
	Up	Down	Up	Down	Up	Down
Flavonoids	103	301	192	216	214	80
Phenolic acids	136	62	47	151	80	32
Terpenoids	51	87	58	63	42	36
Alkaloids	42	72	69	41	77	27
Sum	332	522	366	471	413	175

**Table 2:** In vitro antioxidant capacities of the three *Paphiopedilum* species.

Sample	DPPH (IC_50_, mg/mL)	O_2_^−^· (IC_50_, mg/mL)	OH (IC_50_, mg/mL)
PB	0.194	0.060	0.044
PM	0.204	0.068	0.048
PD	0.490	0.082	0.058

## Data Availability

Data will be made available on request.
